# Rare Case of Lemierre’s Syndrome: *Porphyromonas asaccharolytica*–Induced Monoplegia, Epiduritis, and Meningitis in a 50‐Year‐Old Woman—A Case Report

**DOI:** 10.1155/crdi/5537970

**Published:** 2026-04-19

**Authors:** Alexis Krin, E. Aldige, F. Birot-Jaulin, F. Labaste, S. Buys

**Affiliations:** ^1^ Département d’Anesthésie-Réanimation, Centre Hospitalier Universitaire de Toulouse, Toulouse, France, chu-toulouse.fr; ^2^ Université Toulouse 3-Paul Sabatier, Toulouse, France; ^3^ Service de Réanimation Polyvalente, CH Auch, Allée Marie Clarac, Auch, 32008, France

**Keywords:** epiduritis, Lemierre’s syndrome, peripheral nerve involvement, *Porphyromonas asaccharolytica*

## Abstract

**Introduction:**

Lemierre’s syndrome, characterized by internal jugular vein thrombosis and secondary septic emboli following an oropharyngeal infection, is mainly caused by *Fusobacterium necrophorum*. We report a case of Lemierre’s syndrome caused by *Porphyromonas asaccharolytica*, resulting in pulmonary septic embolism, acute respiratory failure, meningitis, epiduritis, and monoplegia—a complication not previously reported.

**Case Report:**

A 50‐year‐old woman initially presented with tonsillitis and subsequently developed severe sepsis. Clinical examination revealed meningeal stiffness, acute renal failure, elevated inflammatory markers, and severe thrombocytopenia. Cerebrospinal fluid analysis was consistent with meningitis, although cultures remained negative. Following admission, the patient developed acute respiratory failure, requiring transfer to intensive care unit for noninvasive ventilatory support. Computed tomography revealed tonsillar edema, thrombosis of the left internal jugular vein, and bilateral pulmonary consolidations. Empirical treatment was initiated with broad‐spectrum antibiotics (ceftriaxone, metronidazole, piperacillin–tazobactam), platelet transfusions, and intravenous heparin. Blood cultures identified *P. asaccharolytica*, leading to de‐escalation of antibiotic therapy to amoxicillin. Two days later, the patient developed left upper limb palsy. Magnetic resonance imaging showed cervical epiduritis extending from C3 to C7. Corticosteroid therapy was initiated on Day three. The patient was transferred to the medical ward on Day fifteen and discharged from hospital on Day 30. Treatment comprised 4 weeks of corticosteroids therapy and 6 weeks of antibiotic therapy. At follow‐up, partial neurological recovery was observed; however, residual left upper limb paresis persisted. Anticoagulation therapy was maintained for 3 months. Electroneuromyography revealed axonal injury involving the C6‐C7‐C8‐T1 nerve root distribution.

**Conclusion:**

*P. asaccharolytica* is a rare etiological agent of Lemierre’s syndrome. Neurological complications are uncommon but may include meningitis, cerebral abscess, or cranial nerve palsies, the latter being more commonly associated with *F. necrophorum*. We report the case of a 50‐year‐old woman with Lemierre’s syndrome caused by *P. asaccharolytica*, who presented with monoplegia, epiduritis, and meningitis.

## 1. Introduction

Lemierre’s syndrome, characterized by thrombosis of the internal jugular vein (IJV) thrombosis and secondary septic emboli following an oropharyngeal infection [1], is most commonly caused by *Fusobacterium necrophorum*. Its incidence, estimated to range from 0.6 to 2.3 cases per million population [2, 3], is currently increasing [4]. The prognosis remains poor, particularly in cases of delayed diagnosis or treatment, or when neurological complications occur, with reported mortality rates ranging from 6% to 15% [5, 6]. We report the case of a middle‐aged woman with Lemierre’s syndrome caused by *Porphyromonas asaccharolytica*, complicated by pulmonary metastatic septic emboli, acute respiratory failure, meningitis, epiduritis, and monoplegia—a complication that has not previously been described in the literature.

## 2. Case Report

A 50‐year‐old woman with no significant past medical history presented with a two‐day history of sore throat and fever and consulted her general practitioner, who diagnosed viral pharyngitis following a negative rapid streptococcal antigen test. After 1 week of self‐medication with paracetamol and nonsteroidal anti‐inflammatory drugs (NSAIDs), she was admitted to the emergency department. On admission, she reported severe cervical pain and exhibited signs of sepsis, including high‐grade fever (102.2°F), sore throat, dysphonia, and dysphagia. Heart rate was 105 beats per minute, blood pressure 112/68 mmHg, respiratory rate 30 breaths per min, and oxygen saturation 92%. Physical examination revealed meningeal stiffness and crackles at the base of the left lung. An oropharyngeal exam was not feasible because of trismus.

Laboratory investigations demonstrated acute kidney injury (KDIGO Stage 2), a marked inflammatory response, anemia, and severe thrombocytopenia with initially undetectable platelet levels. Arterial blood gas analysis showed hypoxemia and hyperlactatemia. Cerebrospinal fluid analysis revealed clear‐fluid meningitis. Gram stain, antigen testing, and cultures were negative. All biological results are summarized in Table 1.

**TABLE 1:** Notable laboratory data through patient’s hospitalization.

Parameter	Value	Normal range
*Cell blood count*		
White blood cells (×10^9^/L)	13.08	4.0–10.0
Neutrophils, absolute (×10^9^/L)	12.2	1.4–7.7
Lymphocytes, absolute (×10^9^/L)	0.6	1.0–4.8
Monocytes, absolute (×10^9^/L)	0.2	0.2–1.0
Platelets (×10^9^/L)	Undetectable	150–450
Hemoglobin (g/L)	111	120–160

*Hemostasis*		
International Normalized Ratio (INR)	1.01	0.8–1.2
Activated partial thromboplastin time (sec)	34.4	25–35

*Biochemical profile*		
C‐reactive protein (mg/L)	480	< 5
Creatinine (μmol/L)	243	45–84

*Arterial blood gas analysis (4L/min oxygen therapy)*		
PaO_2_ (kPa)	7.59	10–13
PaCO_2_ (kPa)	3.99	4.5–6.0
Lactic acid (mmol/L)	3.11	< 2

*Cerebrospinal fluid analysis*		
Appearance	Clear	Clear
White blood cells (/μL)	314 (80% neutrophils)	< 5
Protein level (g/L)	0.69	0.15–0.45
Gram stain	Negative	Negative
Antigen testing	Negative	Negative
Culture	Negative	Negative

**FIGURE 1:**
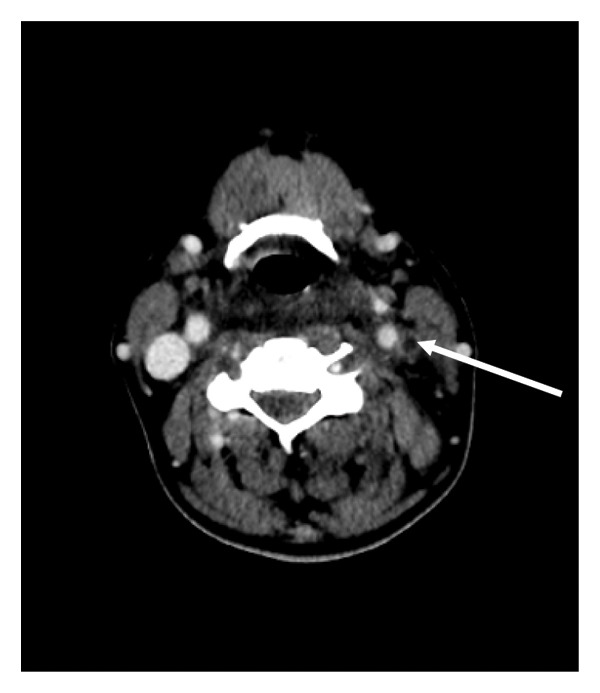
Transverse CT scan. Left internal jugular vein thrombosis.

Six hours after admission, the patient developed acute respiratory failure and was transferred to the intensive care unit (ICU), where noninvasive ventilation was initiated (one‐hour sessions, four times daily). The initial PaO_2_/FiO_2_ ratio was 106, despite a normal chest radiography. Computed tomography revealed tonsillar edema, thrombosis of the left IJV, and multiple bilateral pulmonary consolidations, consistent with septic emboli (Figure 1). Transthoracic echocardiography showed no evidence of valvular vegetation, and myelography was unremarkable.

Empirical treatment was initiated on Day 1 and included intermittent noninvasive ventilation for 10 days and intravenous broad‐spectrum antibiotics: ceftriaxone (2 g infused over 20 min every 8 h), metronidazole (500 mg over 20 min every 8 h, intravenous), and piperacillin–tazobactam (4 g over 20 min every 6 h, intravenous). Two blood cultures grew *P. asaccharolytica*. Triple antibiotic therapy was maintained for five days until the antibiogram, obtained on Day 5, demonstrated high antibiotic susceptibility, allowing de‐escalation to intravenous amoxicillin at meningeal doses (200 mg/kg/day, divided into four daily doses) for 10 days, completing a total of 15 days of intravenous therapy. Oral amoxicillin was subsequently continued at 50 mg/kg/day in three divided doses to complete a 6‐week total duration of antibiotic treatment.

Two platelets’ transfusions were administered on Days 1 and 2 to maintain platelet counts above 10G/L and to perform lumbar puncture. Continuous intravenous unfractionated heparin (400 IU/kg/day) was initiated on Day 3, once spontaneous platelet recovery exceeded 50G/L.

**FIGURE 2:**
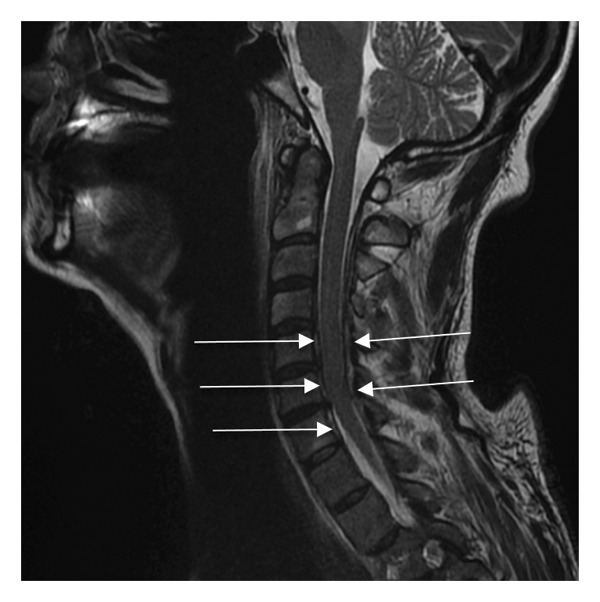
Sagittal MRI. Circumferential epiduritis from C3 to C7.

On Day 3, the patient developed acute left upper limb palsy. Neurological examination revealed edema and a proximal motor deficit predominantly affecting the left shoulder girdle, with muscle strength graded at 1/5 on the Medical Research Council (MRC) scale for arm abduction. Forearm flexion and wrist extension were graded at 2/5 and 3/5, respectively. Sensory examination showed hypoesthesia associated with allodynia, along with abolition of deep tendon reflexes, particularly the bicipital and brachioradialis reflexes. Doppler ultrasonography of the supra‐aortic vessels showed permeable subclavian veins and artery. Magnetic resonance imaging (MRI) excluded brachial plexus compression, spinal or cerebral abscess, extension of the septic thrombosis, and ischemic stroke. However, it revealed circumferential epiduritis extending from C3 to C7 with infiltration of the intervertebral foramina (Figure 2). Corticosteroid therapy was initiated on Day 3 (methylprednisolone, 1 mg/kg/day) and continued for 4 weeks.

The patient was transferred to the medical ward on Day 15 and discharged from hospital on Day 30. At the 45‐day follow‐up, neurological examination showed progressive improvement, although residual left arm paresis with associated muscle atrophy persisted. Venous Doppler ultrasound showed marked regression of the thrombosis with only residual findings in the distal IJV. Anticoagulation therapy was continued for a total duration of 3 months. Electroneuromyography performed on Day 60 revealed axonal damage involving the C6‐C7‐C8‐T1 nerve root territories without evidence of demyelination; a clear distinction between radicular and plexiform involvement could not be established. At the 60‐day follow‐up, moderate residual weakness persisted (MRC grade 4/5 across muscle groups), with reduced deep tendon reflexes. Physical therapy was continued, and the patient was subsequently followed by primary care physician.

## 3. Discussion

The incidence of Lemierre’s syndrome has been increasing in recent decades. After a marked decline during the 1960s and 1970s, its incidence rose from an estimated 0.6–2.3 cases per million to approximately 3.6 cases per million after the 1990s [2, 3]. This resurgence has been attributed to more judicious antibiotic‐prescribing practices [7], as well as increased clinical awareness and improved diagnostic methods [5].

This disease primarily affects young and healthy individuals. NSAIDs have frequently been implicated in the occurrence of Lemierre’s syndrome [8], potentially by masking local inflammatory symptoms and delaying diagnosis, thereby increasing the risk of advanced disease at presentation, including severe sepsis or septic shock.

Lemierre’s syndrome is mainly caused by *F. necrophorum* [3]. In a recent review, *Fusobacterium* spp. were isolated in 415 of 712 cases (58%). Among patients in whom *Fusobacterium* spp. were not detected, *Streptococcus* spp. and *Staphylococcus* spp. were the most frequently identified pathogens. Microorganisms were not specified in 139 patients (18%) [9]. In the present case, blood cultures yielded *P. asaccharolytica*, a Gram‐negative obligate anaerobe. This species is among those most frequently identified association with *F. necrophorum* [10]. *Porphrymonas* species are commensals of the oral cavity, gastrointestinal tract, and female genital tract and are recognized pathogens implicated in septicemia, abscess formation, periodontitis, and endodontic infections [11–14]. In the literature, the *asaccharolytica* strain has been reported in five cases of Lemierre’s syndrome, where it was isolated in pure culture [4, 10, 15–17]. However, despite its isolation from blood cultures in several case reports, the pathogenic role of *Porphyromonas* species remains debated. A synergistic pathogenic complex between anaerobic bacteria and *Fusobacterium* species has been demonstrated, whereby anaerobic organisms facilitate bacterial growth by creating a favorable anaerobic environment [10]. Furthermore, *Porphyromonas* species are frequently identified in polymicrobial infections, often in association with anaerobic cocci or Gram‐negative bacilli.

It has been also postulated that a preceding infection, likely viral or bacterial, may impair local host defenses and facilitate bacterial invasion of the pharyngeal space. Finally, *F. necrophorum* is a fastidious organism, and due to the lower sensitivity of conventional culture methods compared with polymerase chain reaction (PCR), it may not be isolated despite its presence [2, 6, 18].

Platelets were undetectable at admission, without disseminated intravascular coagulation. Thrombocytopenia has been described in infections caused by anaerobic bacteria, including *Porphyromonas* [13]. Several virulence factors have been implicated, notably hemagglutinins that induce platelet aggregation without lysis, as well as phospholipase and lysophospholipase activities contributing to the hemolytic effect [2, 10, 19]. In addition, *Porphyromonas* vesicles possess strong platelet aggregation activity, although the specific active component remains unidentified [10, 20]. Some authors have suggested that the innate immune response and underlying prothrombotic genetic predispositions may partially account for the clinical manifestations of Lemierre’s syndrome [21].

Platelet transfusions were administered with good efficacy to maintain a platelet threshold of 10 × 10^3^/mm^3^ and to allow safe performance of lumbar puncture. Similar use of platelet transfusions in Lemierre’s syndrome has been reported in the literature, despite limited supporting clinical evidence [22, 23]. In consumptive thrombocytopenia, concerns have been raised that exogenous platelet administration may promote thrombosis or adversely affect prognosis. In a large retrospective cohort study, Putake et al. analyzed 41,678 hospitalizations involving consumptive thrombocytopenia. Among these, 11.3% of patients received platelet transfusions and exhibited significantly higher baseline illness severity, including mechanical ventilation or shock. Platelet transfusion was independently associated with increased adjusted odds of mortality [24]. Current international clinical practice guidelines recommend platelet transfusions in adults with consumptive thrombocytopenia without major bleeding when the platelet count is below 10 × 10^3^/mm^3^ [25].

Anticoagulation in Lemierre’s syndrome remains controversial, with favorable outcomes reported both with or without its use. No standardized guidelines exist, and decisions should be based on individual bleeding risk, typically over a 4–6‐week period [3, 5, 26]. Anticoagulation aims to prevent pulmonary septic embolization, and it appears particularly indicated in case of retrograde thrombus extension, thrombosis extension despite effective antimicrobial therapy, or secondary septic localization [27]. Several case reports have documented favorable outcomes in patients with thrombosis managed without anticoagulation, provided that antibiotic therapy was appropriately administered [28, 29].

In a recent literature review, Valerio et al. analyzed 712 cases of Lemierre’s syndrome. The median age of patient was 21 years, 41% were female, and 82% had septic embolism. Among these patients, 363 (56%) received curative anticoagulation therapy [9]. The rate of new or recurrent venous thromboembolism and the incidence of new or worsening peripheral septic lesions were lower in anticoagulated patients. Notably, anticoagulation was not more frequently administered in patients with major bleeding.

A retrospective study including 18 pediatric and adult patients reported that no patient developed recurrent thrombosis or progression during a 3‐month follow‐up period, and all patients showed improvement in thrombi regardless of anticoagulation status. Among the 12 non‐anticoagulated patients, complete and partial thrombus resolution was observed in 9 and 3 patients, respectively, while among the 6 anticoagulated patients, 2 had complete resolution and 4 partial resolution [30]. A meta‐analysis conducted in 2020, involving more than 394 patients, examined the effect of anticoagulation on vessel recanalization (50 patients) and mortality (194 patients). No statistically significant impact of anticoagulation was observed [31]. Similarly, a post hoc observational study of 82 patients with Lemierre’s syndrome, of whom 51 had identified jugular vein thrombosis at diagnosis, found that patients exhibited severe thrombocytopenia, longer time defervescence, and more frequent pulmonary septic emboli. Of these, 20 did not received anticoagulation therapy, 17 received therapeutic doses, and 14 received prophylactic. No significant differences were observed in thrombosis regression, early or late peripheral septic complications, chronic major sequelae, 30‐day mortality, or major bleeding [32].

Finally, a recent retrospective study of 156 patients with septic thrombosis of the IJV found that early initiation of anticoagulation was not statistically associated with improved survival [33]. Despite the lack of robust data, particularly randomized controlled trials, anticoagulation appears relatively safe and may be considered in patients without contraindications.

*F. necrophorum* is commonly resistant to erythromycin and sensitive to penicillin. However, a significant proportion is β‐lactamase positive, and penicillin treatment failures have been reported [34]. Anaerobic species show increasing resistance to penicillin, so metronidazole, which has good activity against *F. necrophorum*, good tissue penetration, including cerebrospinal fluid and rapid clinical improvement, is used [35]. Moreover, due to its bioavailability, it can be administered orally. Due to frequent secondary infections and associated pus collections, monotherapy with anaerobic coverage is not advised; penicillin/b‐lactamase inhibitors in combination with metronidazole are recommended, especially because b‐lactamase‐producing strain of *F. necrophorum* have been reported [35, 36]. Antibiotic therapy should continue. Once infection is controlled, therapy can complete orally and should be continued for at least 6 weeks due to early relapse [2].

Metastatic localizations of Lemierre’s syndrome commonly involve the joints, muscles, bones, liver, skin, spleen, and heart with endocarditis [2, 3, 6]. Neurological complications are relatively rare, accounting for approximately 1.3% of cases and include cerebral or subdural abscesses, stroke, and meningitis [2]. In reported cases of meningitis, blood cultures most frequently *Bacteroides* and *Fusobacterium naviforme*. In the present case, no microorganism was isolated from the cerebrospinal fluid; however, the biological and cytological findings were suggestive of meningitis, possibly due to difficulty in bacterial growth or a sterile inflammatory meningeal reaction.

Epidural and subdural abscesses have only rarely been reported before. Park et al. report the first case of Lemierre’s syndrome involving the epidural space in 2006 caused by *F. necrophorum* [37]. Since then, a limited number of cases have been reported, involving various pathogens, including *Fusobacterium nucleatum* or *Pseudomonas aeruginosa* [38, 39]. Epidural or brain abscesses presumably result from retrograde intracranial extension of IJV thrombosis. These complications appear to occur more frequently in the context of primary otogenic infections than tonsillar infection [36].

In the present case, the patient developed monoplegia, a clinical manifestation not previously described in Lemierre’s syndrome. In contrast, cranial nerve palsies have already been reported, involving Cranial Nerves VI, X‐XI, or presenting as Horner syndrome [6, 40–43]. Potential pathophysiological mechanisms include posterior compartment invasion, neurotoxic process, or severe meningeal infection. Syed et al. described a case of Lemierre’s syndrome involving Cranial Nerves IX, X, XI, and XII associated with Horner syndrome, without identification of a causative pathogen; neurological recovery was not reported. Blessing et al. reported a patient who developed secondary hypoglossal nerve palsy due to *F. necrophorum*. Brain MRI showed inflammatory lesions near the hypoglossal canal without evidence of retropharyngeal abscess, suggesting toxic neuritis as a potential mechanism. Following combined surgical management, appropriate antibiotic therapy, and curative anticoagulation, near‐complete neurological recovery was observed [40].

In general, neurological recovery in Lemierre’s syndrome is often prolonged, with patients either achieving gradual improvement or retaining minor sequelae. Another reported case of Lemierre’s syndrome without pathogen identification described involvement of the VI and XII cranial nerves with complete recovery of the hypoglossal function and partial recovery of the abducens nerve function after several weeks [41].

To our knowledge, this report describes the first case of meningitis and epiduritis with left upper limb palsy due to *P. asaccharolytica*, and the first documented case of Lemierre’s syndrome presenting with monoplegia. We hypothesize a toxic neuritis with involvement of the brachial plexus. In addition to effective antimicrobial therapy, corticosteroid treatment was initiated despite the absence of robust scientific evidence supporting its use in this context. Notably, in previously reported case of cranial nerve involvement, corticosteroid therapy was not mentioned, yet neurological recovery was often nearly complete.

This case report has several limitations. First, *P. asaccharolytica* was the only microorganism identified despite repeated blood cultures. Although the strain *asaccharolytica* has been reported as the causative agent in five previously published cases of Lemierre’s syndrome, *F. necrophorum* may remain undetected despite its presence, particularly in the absence of PCR testing.

Second, therapeutic‐dose anticoagulation was initiated in view of the severity of the clinical presentation and the presence of multiple septic pulmonary emboli. However, given the absence of standardized guidelines, anticoagulation was started only after the platelet count exceeded 50 × 10^3^/mm^3^ of platelets in order to minimize the risk of bleeding.

Third, antimicrobial therapy was switched to amoxicillin on Day 5 based on the high susceptibility profile of *P. asaccharolytica*. This change may have resulted in incomplete antimicrobial coverage of *F. necrophorum* had it been present.

Fourth, the patient was discharged from hospital on Day 60, and neurological follow‐up was not continued.

Finally, we hypothesize that the patient’s monoplegia resulted from a toxic neuritis with plexus involvement. This hypothesis is supported by MRI findings, which excluded brachial plexus compression of the brachial plexus as well as subdural or parenchymal abscess formation.

In conclusion, we present a rare case of Lemierre’s syndrome caused by *P. asaccharolytica*. Although neurological complications are uncommon in Lemierre’s syndrome and are most frequently associated with *F. necrophorum*, this report describes, to our knowledge, the first case of limb palsy associated with Lemierre’s syndrome regardless of the causative organism, and the first case of neurological involvement characterized by meningitis, epiduritis, and palsy due to *P. asaccharolytica*.

This case underscores the critical importance of early recognition and prompt, aggressive management of Lemierre’s syndrome. A multidisciplinary approach combining appropriate antimicrobial therapy and, when indicated, anticoagulation may contribute to favorable outcomes, as illustrated by the patient’s gradual neurological improvement. Lemierre’s syndrome should be considered even when *F. necrophorum* is not isolated, as the diagnosis cannot be excluded solely on the basis of neurological manifestations.

**Nomenclature**
ICU: Intensive care unit
IJV: Internal jugular vein
MRC: Medical Research Council
MRI: Magnetic resonance imaging
NSAIDs: Nonsteroidal anti‐inflammatory drugs
PCR: Polymerase chain reaction

## Author Contributions

Alexis Krin wrote the case report. F. Birot‐Jaulin, S. Buys, F. Labaste, and E. Aldige have contributed to the conception of the case report.

## Funding

The authors report no funding for this case report.

## Ethics Statement

Ethics committee of Auch Hospital agreed to the completion and drafting of this case report.

## Consent

The patient gave his written and oral consent for the publication of the case and accompanying images.

## Conflicts of Interest

The authors declare no conflicts of interest.

## Data Availability

The data that support the findings of this study are available from the corresponding author upon reasonable request.
